# Changes in testicular histomorphometry and ultrastructure of Leydig cells in adult male Japanese quail exposed to di (n-butyl) phthalate (DBP) during the prepubertal period

**DOI:** 10.1007/s11356-023-25767-2

**Published:** 2023-03-09

**Authors:** Umar M. Bello, Mary-Cathrine Madekurozwa, Hermanus B. Groenewald, Augustine Arukwe, Tom A. Aire

**Affiliations:** 1 Department of Anatomy and Physiology, Faculty of Veterinary Science, University of Pretoria, Private Bag X04, Onderstepoort, 0110 South Africa; 2 Laboratory of Cell Biology and Histology, Department of Veterinary Anatomy, Faculty of Veterinary Medicine, Ahmadu Bello University, Zaria, Nigeria; 3 Department of Biology, Norwegian University of Science and Technology (NTNU), Høgskoleringen 5, 7491 Trondheim, Norway; 4 Department of Anatomy, Physiology and Pharmacology, School of Veterinary Medicine, St. George’s University, True-Blue, St. George’s, Grenada

**Keywords:** Di(n-butyl) phthalate, Male Japanese quails, Leydig cell, Ultrastructure, Microstereology, Endocrine disruption

## Abstract

Phthalate esters, such as di(n-butyl) phthalate, (DBP), are synthetic chemical pollutants commonly used as plasticizers in the manufacture of plastics. In the present study, we investigated the effects of DBP in the testes of adult male quails (*Coturnix cortunix japonica*) exposed by oral gavage to variable doses of DBP (0 [control], 1, 10, 50, 200, and 400 mg/kgbw^−d^), for 30 days during the prepubertal period, using histo-morphometric and ultrastructural techniques. Generally, significant decreases in seminiferous tubular diameter (STD) and epithelial height (SEH) were observed predominantly at the highest DBP doses (200 and 400 mg/kg), as compared to medium (50 mg/kg), and lowest doses (1 and 10 mg/kg) as well as the control group. Ultrastructurally, apparent dose-specific degenerative changes were observed in the Leydig cells. The lowest DBP doses (1 and 10 mg/kg) did not produce significant effects on Leydig cell ultrastructure, whereas, at the highest doses (200 and 400 mg/kg), the Leydig cells were remarkably conspicuous in the interstitium and appeared foamy. There was a preponderance of electron-lucent lipid droplets which crowded out the normal organelles of the cell, as well as increases in the number of dense bodies in the cytoplasm. The smooth endoplasmic reticulum (sER) was less obvious, compacted, and wedged between the abundant lipid droplets and mitochondria. Taken together, these findings indicate that pre-pubertal exposure of precocious quail birds to DBP, produced parameter-specific histometric tubular changes, as well as dose-dependent cyto-structural derangement of the Leydig cells; which consequently may lead to overt reproductive impairments in the adult bird in the environment.

**Graphical Abstract:**

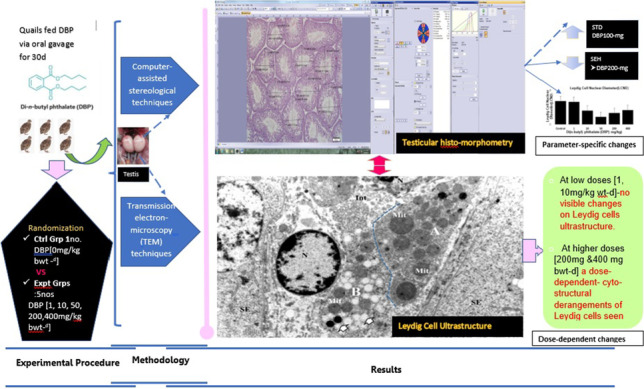

## Introduction

Components used in plastics, such as phthalate esters (PEs) are present in most consumer products including children’s toys and body care products (Hubinger and Havery 2006; Schettler 2006; Andrady and Neal 2009; Perico et al. 2022). PEs are also known as endocrine-disrupting compounds (EDCs) owing to their ability to modulate the endocrine system and thereby cause adverse effects on reproductive processes in humans and wildlife species in the environment (Diamanti-Kandarakis et al. 2009). Numerous wildlife species and humans are currently exposed to a wide variety of potential endocrine disrupters (ED), and at concentrations that produce varying effects due to species differences in the endocrine regulation of reproduction (Ito et al. 2005; De Lange et al. 2009). In most mammalian models, PEs, including di (n-butyl) phthalate (DBP), are known to induce testicular injury and adversely affect testicular differentiation and spermatogenesis by provoking germ cell loss, altered Leydig cell function and testicular atrophy (Ge et al. 2007; Spade et al. 2015; Walker et al. 2021).

Spermatogenesis represents a complex and dynamic process, and its development is hinged on precisely timed events (Hess and Franca 2009). It is well established that spermatogenesis is sensitive to environmental toxicants, particularly EDCs (Alam et al. 2010a; Yeung et al. 2011; Moody et al. 2013; Jenardhanan et al. 2016). Several EDCs affect reproductive function by inducing apoptosis in germ cells, thereby causing defective spermatogenesis (Cheng et al. 2011; Wan et al. 2013). Testicular toxicity resulting from exposure to PEs is age-dependent, with studies conducted on rats having shown that testicular damage due to PE exposure is more severe in sexually immature (pre-pubertal) than in mature adult male rats (David et al. 2000; Dalgaard et al. 2001; Hannas et al. 2011). Exposure of adult male rats to high doses of certain phthalates resulted in rapid and severe changes in the testis (Dalgaard et al. 2001). In testicular tissues, the primary cellular target of phthalate-induced exposure are Sertoli and/or Leydig cells, which exhibit biochemical and morphological changes (Moffit et al. 2007; Shirai et al. 2013; Wakui et al. 2013; Bello et al. 2014; 2019).

There are few in vivo or in vitro reproductive studies which characterize the adverse effects of phthalate-induced exposure, especially at the ultrastructural level (Andriana et al. 2004a, b; Tay et al. 2007; Shirai et al. 2013; Qin et al. 2018). Therefore, the characterization of chemically-induced cellular alterations, using various morphologic tools, has been shown to be a valid approach for assessment of the deleterious effects of environmental toxicants on organs and tissues (Creasy 2003), with a view to gaining a better insight into the mechanisms through which phthalate exposure causes tissue damage.

Despite extensive research into environmental influences on male reproductive health, the scope of the problem is largely unclear, and specifically, the reproductive health implications of phthalate-induced exposure are not well documented. In general, the mechanisms of toxicity of phthalates at the biological level, are either poorly understood or unknown. Furthermore, studies on the adverse effects of phthalate esters on avian male reproduction, with regard to precise histo-morphometric and ultrastructural evaluation of the seminiferous tubule, are scanty. It is possible that xenobiotic compounds, such as DBP, may induce different effects in birds than in mammals (Ottinger et al. 2005).

Therefore, the aim of the present study was to investigate the seminiferous tubular (histometric) and Leydig cell ultrastructural (subtle) changes in the testis of adult Japanese quail (*Coturnix coturnix japonica*) following a 30-day (repeated) exposure to di (n-butyl) phthalate DBP at pre-pubertal period, using histometric and ultrastructural techniques. It is hoped that the data obtained in this study would provide a clear morphological evidence of the detrimental effects of endocrine-disrupting properties of the environmental toxicant (DBP), in this specie.

## Materials and methods

### Chemicals

Di (n-butyl) phthalate (DBP) [CAS Number 84–74-2, technical grade, 99% purity] was purchased from Sigma-Aldrich (Pty) Ltd. (Johannesburg, South Africa). All other reagents were of the highest commercially available grade.

### Animals, experimental design, and dosing considerations

Ninety (90) pre-sexed, 6-week-old male Japanese quails, *Coturnix coturnix japonica* weighing (180-200 g), procured from the Poultry section of the Irene Animal Improvement Research Station, Gauteng Province, Pretoria, were used for this study. The animals were housed, until 10 weeks of age, in battery cages with a dimension of 49 × 95 × 51 cm, in a well-ventilated room maintained at standard temperature (25 ± 2 °C), relative humidity of (25 ± 5%), and controlled photoperiod of 16L:8D light/dark cycle. (SANS Guidelines 2008). Throughout the experimental period, the animals were fed on a special (i.e., phthalate/bisphenol-A (BPA)-free), high-protein diet (ObaroFeeds™, Pretoria, South Africa), with drinking water provided ad libitum.

The experiment was designed in accordance with the avian toxicity testing studies (OECD Guidelines 2010). The animals were randomly divided into six groups (*n* = 15) with individuals exposed by oral gavage to different doses of DBP dissolved in corn oil (at 0 [control], 1, 10, 50, 200, and 400 mg/kg body weight), once daily for 30 consecutive days. The control group received only the corn oil base. These DBP doses were chosen based on previously published study (Bello et al. 2014); and to test the effects of *environmentally-relevant* (low) doses (i.e., 1–50 mg/kg) and doses that are regarded at the level of extreme and acute exposure conditions (i.e., 200 and 400 mg/kg). Further justification for the choice of doses was also based on the fact that the testicular cyto-morphological effects of DBP in avian species are limited and generally lacking in the literature, thereby warranting the use of a broad range of doses. In the rat model, for instance, the *no-observed-adverse-effect-level* (NOAEL) of DBP by intra-gastric lavage was 50 mg/kg/day (Mylchreest et al. 2000; Zhang et al. 2004); while a dose of 0, 15, and 35 µg DBP/L (Aoki et al 2011) and 0.1, 0.5.1.0, 5.0, or 10 ppm DBP (Lee and Veeramachaneni 2005) have been reported in fish and amphibians, respectively. Therefore, the choice of tested doses used in the present avian study was aimed to span possible *environmentally-relevant* concentrations, as well as to achieve a *dose–response* relationship of DBP exposure approach, useful for developing a mechanistic model that incorporates cellular responses in adult quail testis following pre-pubertal exposure to various DBP dose levels. After the last administration of DBP, the experimental and control animals were sacrificed, using an overdose of carbon dioxide (CO_2_) inhalation anesthesia. The testes were quickly excised and blocks of tissues were fixed for light (histological) and transmission electron microscopy techniques (TEM), as described below.

### Transmission electron microscopy (TEM) procedure

Small blocks (i.e., 1 mm^3^) of testicular tissue were taken from each bird (control and DBP-treated groups); (*n* = 5 per group), and were immediately fixed by immersion in small Eppendorf™ tubes containing 4% glutaraldehyde in 0.13 M Millonig’s phosphate buffer at pH 7.4, for at least 24 h. The samples were then post-fixed in similarly buffered 1% Osmium tetroxide for 2 h, dehydrated in a series of graded ethanol concentrations, and embedded in epoxy-resin at a ratio of 1:2 for 1 h, 1:1 for 2 h, and 100% resin overnight. For each bird, three separate tissue blocks from each testis were prepared for microtomy. Semi-thin sections, of 1 µm thickness, were cut with a diamond knife and stained with toluidine blue. Stained sections were photographed with a DP 72 camera mounted on Olympus BX 63 microscope (Olympus Corporation, Tokyo, Japan). Ultra-thin (50–90 nm) sections of selected areas were cut on a Reichert-Jung Ultracut (C. Reichart AG, Vienna, Austria) using a diamond knife, collected onto copper grids, and stained with Reynold’s lead acetate and counterstained by using an aqueous saturated solution of uranyl citrate (Ayache et al. 2010). The sections were examined in a Phillip CM 10 transmission electron-microscope-TEM (Phillips Electron Optical Division, Eindhoven, Netherlands), operated at 80 kV. A mega view III side-mounted digital camera (Olympus Soft Imaging Solutions GmbH, Munster, Germany) was used to capture the images, and iTEM software (Olympus Soft Imaging Solutions GmbH, Münster, Germany) to adjust the brightness and contrast.

### Histological and micro-stereological procedures

For light microscopic observations, testicular samples from DBP-treated and control groups were immediately fixed in 10% Neutral Buffered Formalin (NBF) for 24 h, dehydrated in ascending grades of ethanol, and embedded in paraffin; sectioned at 5 µm, and stained with hematoxylin and eosin (H&E), and subsequently examined under an Olympus BX 63® (Olympus Corporation, Tokyo, Japan) microscope.

#### Micro-stereological evaluation of the seminiferous tubular epithelium

Quantitative measurements of the seminiferous tubule were done on forty-two (42) H and E-stained histological sections per testis, using a stereological module of computer-assisted digital image analyzer (*CellSens®Dimension* ver 1.6 software program) running on a digital computer. The digitized images were acquired, using an Olympus BX63® (Olympus Corporation, Tokyo, Japan) microscope, fitted with an Olympus DP72 camera. The *CellSens® Dimension* stereological module works in tandem with the *CellSens® Dimension* Multiple Image Alignment (MIA) tools and was used to facilitate the creation of high-resolution, *panoramic* digital images, capable of covering the complete microscope stage. In this way, sequential imaging across different geometric parameters and/or areas of a specimen were possible. Briefly, sections of testicular tissue were examined at low magnification (10 ×), and then at high magnification (100 ×) for more detailed analyses of seminiferous tubular structure, as described by (Romano et al. 2010). These included analyzing the linear morphometry of the following parameters: seminiferous tubular diameter (STD), seminiferous epithelial height (SEH), seminiferous luminal diameter (SLD), and the area of the seminiferous tubule (AST).

In estimating STD, morphometric measurements of the seminiferous tubular epithelium were taken from one end of the basal lamina to another, the SEH measurements were taken as the linear length of the seminiferous tubule from the boundary layer (basal lamina) to the luminal edge, while SLD, were calculated as the longest measurement from one luminal edge to the other, while the cross-sectional areas of seminiferous tubules of circular transverse sections of seminiferous tubules were taken for each group (*n* = 7), as the AST measurements. For each seminiferous tubule parameter, measurements were performed on, at least, 20 round or nearly round tubular profiles, chosen randomly in each microslide (*n* = 7)/group. With the aid of a digitized mouse, at least three measurements were made, for each parameter, in each animal group. Captured data (digitized images) were automatically calculated by interactively sketching each of the transversely sectioned seminiferous tubule measurements and then averaged. These parameters were sequentially determined using a systematic, random sampling scheme (Gundersen and Jensen 1987; Cruz-Orive 1993), which allowed for an unbiased numerical estimation of the parameters.

### Statistical analysis

All the micro-stereological data generated were expressed as mean ± standard error of the mean (SEM). After normal students’ *t*-testing for the homogeneity of variance in the dataset, a one-way analysis of variance (ANOVA) was applied to evaluate the differences between treatments for each parameter. Subsequently, the data were subjected to Duncan’s multiple comparison tests, used to make a comparison between DBP treatment groups, when and where appropriate. All statistical analyses were carried out, using Statistical Product and Service Solutions (SPSS) for Windows, 19th edition (IBM, IL, USA). The value of *p* < 0.05 was considered significant.

## Results

### Effects of DBP on Leydig cell morphology

**Fig. 1:**
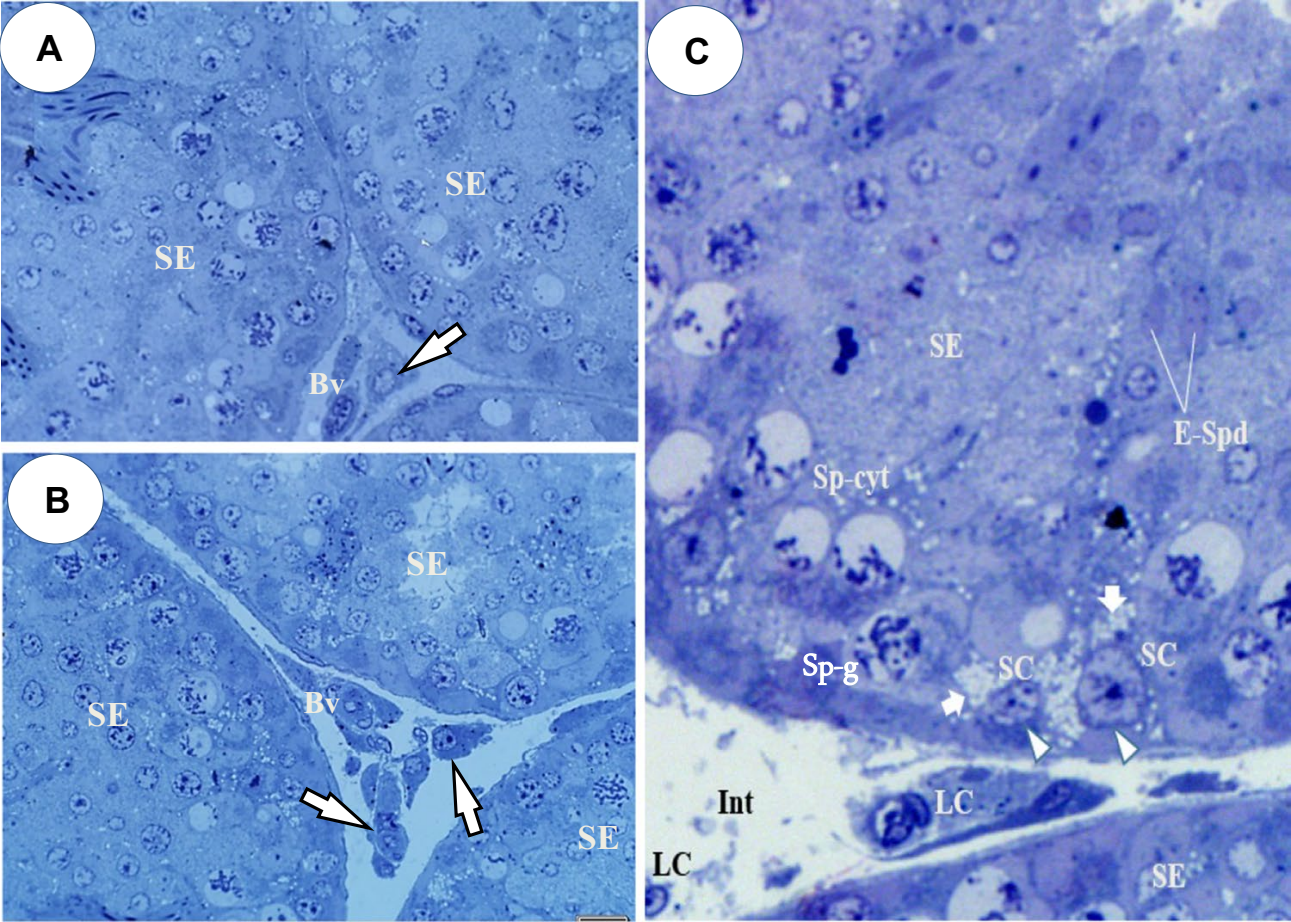
Photomicrograph of toluidine blue-stained (semi-thin) sections of the interstitium of adult quail testis taken from **A** DBP (0 mg/kg) control or low dose (DBP (1, 10 mg/kg) group and **B** medium-dose (DBP 50 mg/kg) groups with isolated masses of Leydig cells (white arrows) found in close association with blood vessels (Bv), and displaying a few lipids. Spermatogenesis appears normal. **C** is a high-powered view of the DBP-treated (200 mg or 400 mg/kg) group. Note the abundance of lipid droplets in the basal part of Sertoli cell cytoplasm (white squat arrows), but rare in the adluminal compartment (white arrowhead ). The intertubular space (Int) shows Leydig cell cytoplasm filled with numerous lipid droplets. Seminiferous epithelium (SE), Spermatogonia (Sp-g), Spermatocytes (Spt-cyt), Sertoli Cell (SC) and elongated spermatids (E-Spd) at various stages of differentiation. Toluidine blue stain (× 40)

**Fig. 2:**
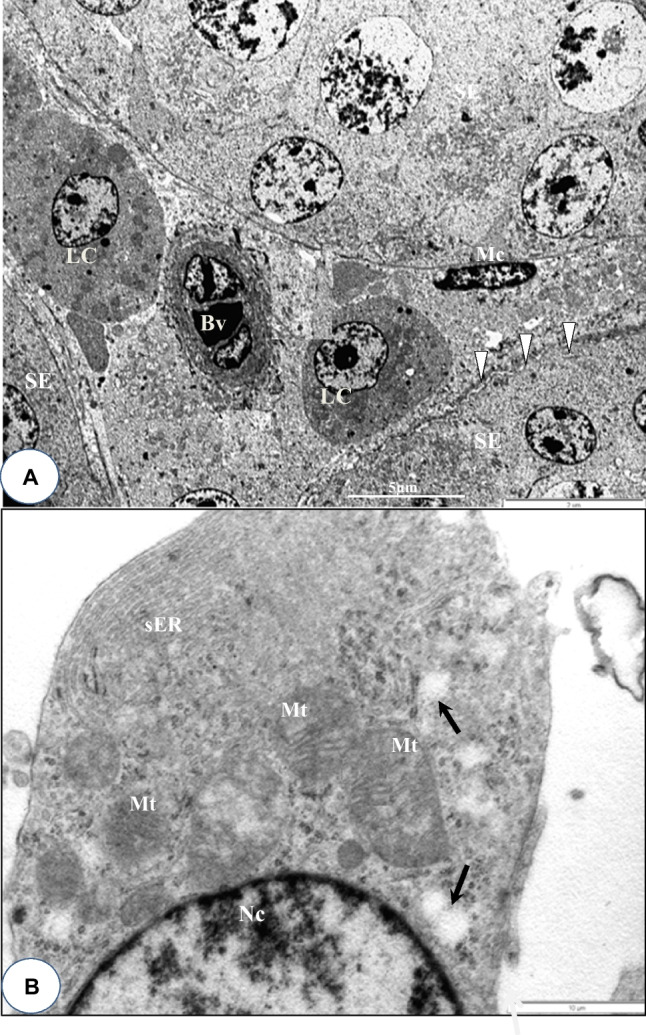
**A** and **B** Transmission electron micrograph of the testis of DBP-(0 mg/kg) control or low doses DBP (1, 10 mg/kg) showing parts of seminiferous tubules and normal Leydig cells (LC) seen in the Interstitium. Seminiferous epithelium (SE), blood vessel (Bv), myofibroblast (Mc), boundary tissue (arrow heads). **B** High power view of part of a normal Leydig cell showing numerous mitochondria (Mt), abundant smooth endoplasmic reticulum (sER), a few lipid droplets (black arrows) and a mildly heterochromatic nucleus (Nc) Bar in Fig. 2A (5 µm), Bar in Fig. 2B (10 µm)

**Fig. 3:**
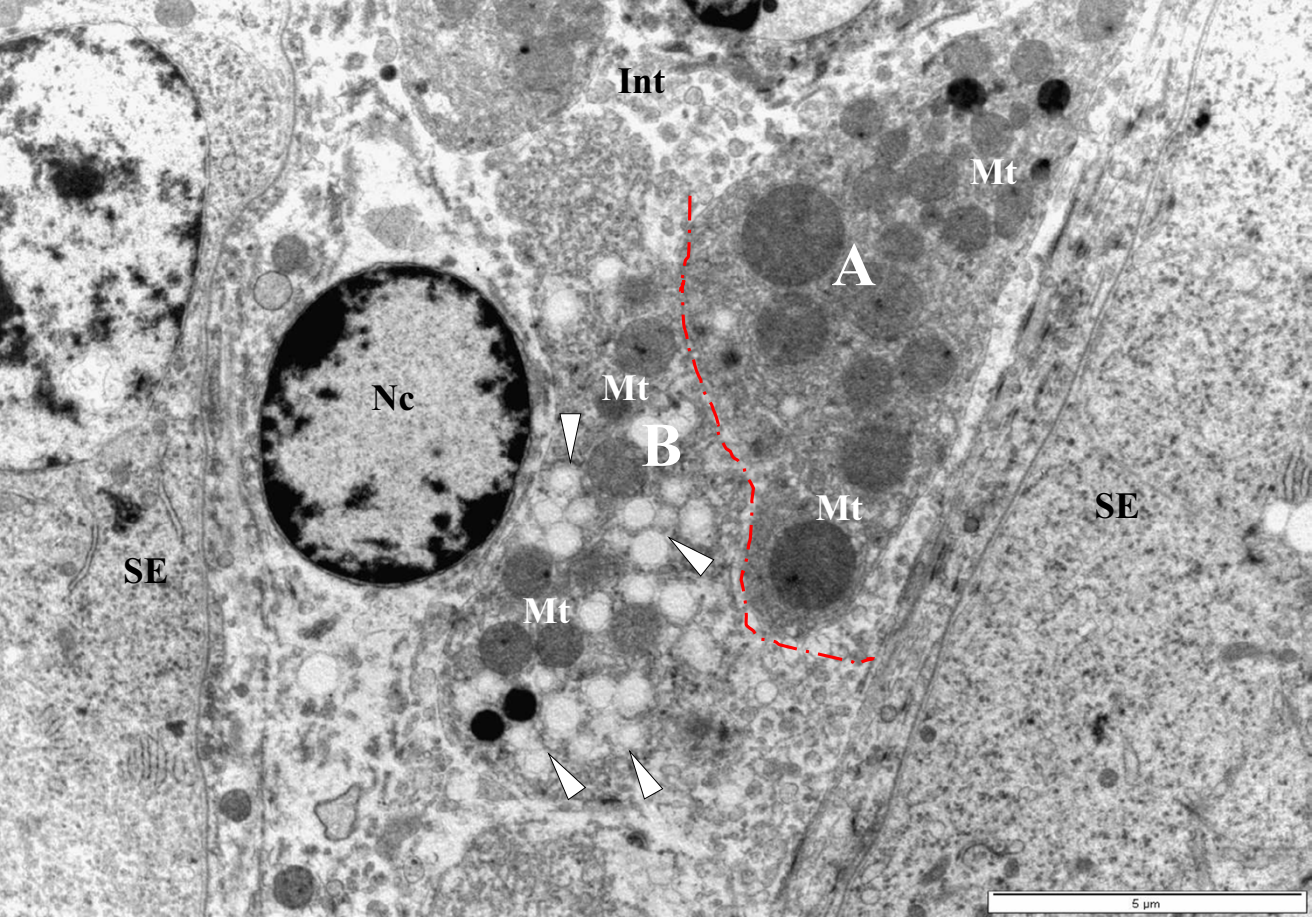
Electron micrograph section of the interstitium (Int) of the quail testis taken from DBP medium-dose (50 mg/kg) group. With the aid of a free-form (red, dash-line) demarcation, Leydig cell **A** appears normal, while the other, **B**, shows an abnormal accumulation of intracellular lipid droplets (white arrowheads), which crowded out normal organelles. The mitochondria (Mt) in both cells are, generally, of different sizes. Seminiferous epithelium (SE), Nucleus (Nc) Bar = 5 µm

**Fig. 4:**
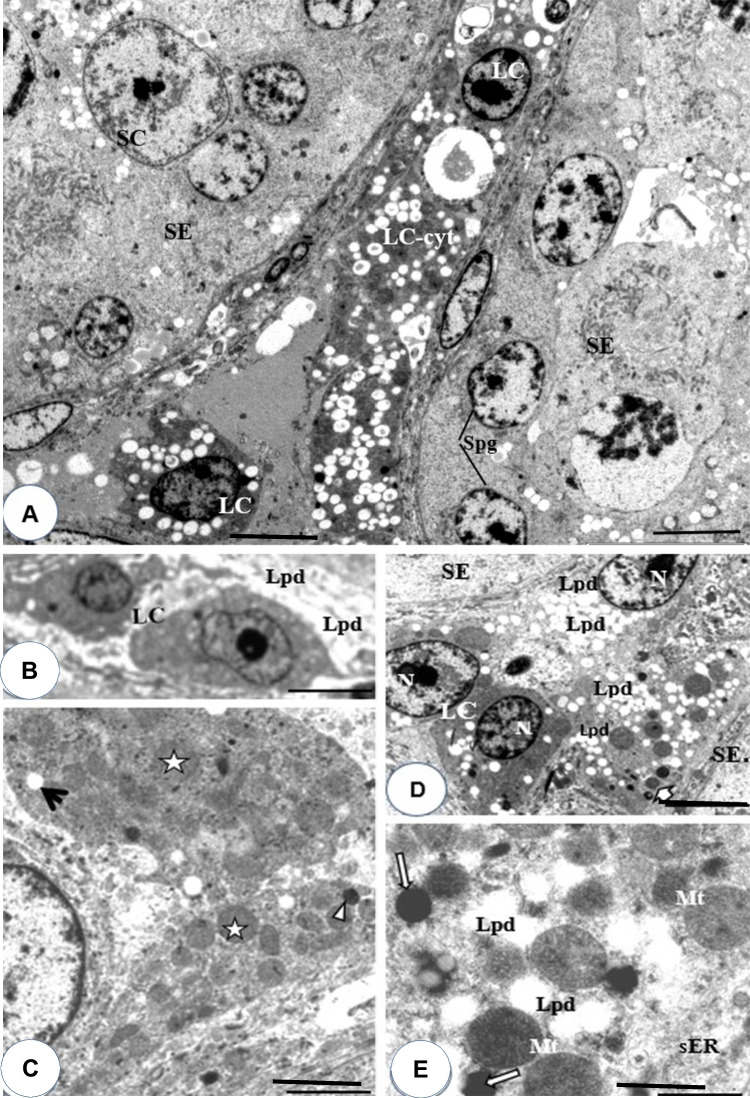
**A** Survey electron micrograph of the testicular interstitium taken from a DBP high-dose (200 and 400 mg/kg) groups, displaying numerous, “foamy” Leydig cells (LC). **B** is high-powered view of (**A**) showing two neighboring Leydig cells (LC) with indented nuclei, due to lipid droplets (Lpd) in the interstitium. **C** is a high-powered view of a Leydig cell displaying numerous mitochondria (white asterisk), very few lipid droplets (black arrow) and dense bodies (arrowhead) in the cytoplasm. **D** and **E** (high-power view) are parts of Leydig cells, exhibiting numerous lipid droplets (Lpd) in the interstitium. In **E**, aside from the preponderance of lipid droplets, the dilated smooth endoplasmic reticulum (sER) is squashed between other organelles and abundant inclusion bodies (white arrow). Mt, mitochondria; Lpd, Lipid droplets; Spg, spermatogonia; SC, Sertoli cell; LC, Leydig cells; LC-cyt, Leydig cell cytoplasm; SE, seminiferous epithelium. Bars: **A** and **D** = 10 µm; **B**, **C**, and **E** = 5 µm

Figure 1A–C depicts the toluidine-blue (semi-thin) transverse sections of adult quail testes at various dosage regimes, as viewed under the bright-field microscopy. In quail testis fed by intragastric gavage with DBP (0 mg/kg) or low/median doses (1, 10, 50 mg/kg), the interstitium (Fig. 1A, B), showed isolated masses of normal Leydig cells with few lipids droplets. However, at high DBP doses (200 mg- and 400 mg/kg), the Leydig cell cytoplasm increasingly displayed aggregations of lipid droplets (Fig. 1C). Ultrastructurally, there were no evident morphological abnormalities in Leydig cells of adult quails fed with corn oil (control) or lowest doses of DBP (1 and 10 mg/kg) during the prepubertal period (Fig. 2A and B). However, in the medium (50 mg/kg) DBP dose group, apparently normal Leydig cells were seen together with abnormal Leydig cells in the interstitium (Fig. 3A). The latter (Fig. 3B) displayed numerous lipid droplets which crowded out the normal organelles of the cell, such as mitochondria and smooth endoplasmic reticulum (sER). On the other hand, significant changes in Leydig cells were observed in the highest (200 or 400 mg/kg) DBP dose groups. The Leydig cells were conspicuous in the interstitium because they appeared foamy (Fig. 4A). Some Leydig cell nuclei were irregular in shape because of lipid droplets that indented them (Fig. 4B), compared to the control group (Fig. 2A). In the neighborhood Leydig cells (Fig. 4C), there was an increase in the number of mitochondria (white asterisk) and dense bodies; as well as a preponderance of lipid droplets in the cytoplasm (Fig. 4D). In addition, the smooth endoplasmic reticulum (sER) was less obvious, and wedged between the abundant lipid droplets and mitochondria (Fig. 4E).

### Effects of DBP on seminiferous tubulo-morphometry

**Fig. 5:**
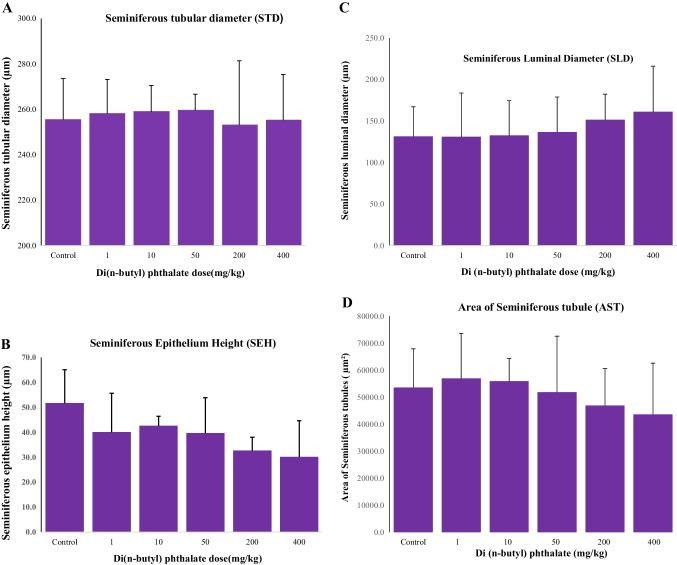
The effects of 30-day dietary exposure (pre-pubertal) to different dose regimen of di(n-butyl) phthalate (DBP) on histometric parameters: **A** seminiferous tubular diameter (STD), **B** seminiferous epithelial height (SEH), **C** seminiferous luminal diameter (SLD) and **D** the area of seminiferous tubules (AST)

In the current study, Fig. 5A-D, shows the graphical summaries of morphometric data of various testicular (i.e., STD, SEH, SLD, and AST) parameters, evaluated in both control and DBP-treated groups. Relatively, there was no significant change (*p* > 0.05) in STD values between low and medium (1, 10, 50 mg/kg) DBP-treated and control groups, but there was a slight decrease (*p* < 0.05) in STD at the highest (200 and 400 mg/kg) DBP dose groups, when compared to the control vehicle group (Fig. 5A). In contrast, the seminiferous tubules of all DBP-exposed quails showed apparent dose-specific reductions in germinal epithelial height. Furthermore, the maximum reduction in SEH occurred at the highest (200 and 400 mg/kg) DBP dose groups, compared with the controls, while the epithelial height in the medium (50 mg/kg) and lowest (1 and 10 mg/kg) DBP treatment groups were slightly reduced, relative to the control group (Fig. 5B). On the other hand, there was a slight but progressive increase (albeit not significant, *p* > 0.05) in SLD values, from low to the highest DBP dose levels (Fig. 5C), as compared to DBP control groups. AST values decreased as the dose level of DBP increased, such that the AST was highest at (1 mg- and 10 mg/kgbwt) DBP groups and lowest at (200 mg- and 400 mg/kgbwt) DBP dose groups (Fig. 5D).

## Discussion

It was previously demonstrated that PEs, including DBP, are anti-androgenic environmental contaminants that cause adverse biological effects on male reproductive health, growth, and development in both human and wildlife (Foster et al. 2001; Oehlmann et al. 2009; Alam et al. 2010a; Bello et al. 2014, 2019). Since reproductive development is a continuous process throughout ontogeny in vertebrate species, it is susceptible to changes in physiology due to exposure to environmental contaminants at different stages of development, including structural differentiation and hormonal synthesis (Diamanti-Kandarakis et al. 2009). In addition, there has been considerable difficulty in creating universally accepted and reliable end-point(s) for exposure of avian species to EDCs due to a vast array of reproductive strategies. However, mammalian, reptilian, and piscean data have provided valuable insights on the likely mechanisms of action of EDCs. The impairment of testicular development as a result of PE exposure has been shown to be age-dependent, with mature animals being less sensitive than immature animals (Dalgaard et al. 2001). Although previous studies have shown compromised male copulatory behavior in the Japanese quail after exposure to EDCs, and, thus, providing a reliable and sensitive indicator of embryonic gonadal hormone exposure (Adkins 1979), few studies have investigated ultrastructural and morphometric changes in the testis following exposure to environmental contaminants. Therefore, the present study is unique, because it provides valuable information on PEs-mediated alterations of testicular variables and that may have consequences on avian reproductive health.

### Effect of pre-pubertal exposure to DBP on the ultrastructure of Leydig cell

The Leydig cells, exclusively, are the primary sites of testicular androgen production in males (Griswold and Behringer 2009; Shima et al. 2013) Androgens are essential hormones necessary for the regulation of spermatogenesis. In this study, we observed that Leydig cells in the highest DBP (200 and 400 mg/kg) dose groups showed marked cyto-morphological changes, resulting in reduced cell size, compared with other treatment groups or controls. Our findings were in accordance with previous observations on the ultrastructural changes that included diminished amount of sER and a significant reduction in Leydig cell size, due to nuclear shrinkage and hyper-chromatization as reported by Blanco et al. (2010) in rodents. In our present study, Leydig cells in the DBP-exposed groups were characterized by a preponderance of electron-lucent lipid droplets which crowded out the normal organelles of the cell. Interestingly, the observed DBP-induced aggregation of lipid droplets in the cytoplasm may be indicative of either an increased lipid synthesis (lipidosis) or reduced utilization of lipids, and more specifically, cholesterol, by the cells. Increased synthesis and accumulation of lipid droplets in testes have been observed in pre-pubertal rats exposed to DBP (Alam et al. 2010b). Previously, Bell (1982) demonstrated that in certain tissues, di-(2-ethylhexyl) phthalate [DEHP] inhibited fatty acid synthesis. Lipid droplets in Leydig cells are generally rich in cholesteryl esters and serve as the reservoir of cholesterol for testosterone synthesis (Fujimoto and Parton 2011; Kraemer et al. 2013; Shen et al. 2016) and, hence, they may increase in number and/or size when the synthesis of testosterone is inhibited.

The exact mechanism by which DBP exposure produced lipidosis, as observed from the present study, is not well understood. However, considering that Leydig cell steroidogenesis begins with intracellular molecular trafficking (transport) of de-esterified substrate cholesterol from the lipid droplets into mitochondria (Hales et al. 2005; Manna et al. 2013; Tarique et al. 2019), it is probable that PEs, including DBP, inhibit steroid production by acting at the level of cholesterol transport across mitochondrial membranes, resulting in the accumulation of lipid droplets in the cytosol of the Leydig cells (Hu et al. 2010; Savchuk et al. 2015). In this context, the present study has demonstrated that a 30-day exposure of precocious Japanese quail testis to DBP, during the prepubertal period, could alter spermatogenesis in adult birds, especially at high dose levels. The main changes/effect being are cyto-morphological alterations in the Leydig cell, which changes could lead to deranged spermatogenesis and, consequently, infertility in this species. It is suggested that future studies are designed to involve more specific and in-depth indicators of degenerative changes such as apoptosis, along with other cyto-morphological approaches in the elucidation of biochemical events responsible for DBP anti-androgenic effects in the quail testis.

### Effect of DBP on seminiferous tubular-histomorphometry

Several qualitative (Alam et al. 2010a; Ahbad and Barlas 2013; Moody et al. 2013; Shirai et al. 2013; Shono and Taguchi 2014) and quantitative (Auharek et al. 2010; Wakui et al. 2013) studies have been performed in order to detect testicular toxicity due to PE exposure in animals. Relatively, quantitative studies on the seminiferous tubule epithelium have received very little attention. Recent reports have shown altered testicular differentiation following prenatal and/or post-natal exposure to DBP (Alam et al. 2010a; Jobling et al. 2011; Ahbad and Barlas 2013; Giribabu et al. 2014). The STD is used as an important, functional evaluation of spermatogenetic activity in experimental and toxicological assays. In the present study, a significant reduction was observed in the STD of quails treated with high doses (200 and 400 mg/kg) of DBP, as compared to low doses or control groups. The decrease in tubular diameter with the higher dose of DBP was possibly due to the reduction of secretion of seminiferous tubular fluid. Alternatively, it could be due to cell death (apoptosis) or extensive sloughing of germ cells, as has been shown in rats and mice (Alam et al. 2010a; Zhu et al. 2010). According to Nakai et al. (1992), sloughed germ cells can block the efferent ductules and, consequently, decrease the tubular diameter due to fluid back-up. It has been established that there is a strong positive relationship between the seminiferous tubular diameter and spermatogenesis (Franca and Russell 1998; Franca and Godinho 2003). Nair et al. (2008), in Wistar rats, also reported that DBP exposure produced dose-dependent reproductive toxicity due to significant reductions in STD.

The current study has further revealed that the SEH was significantly reduced in all DBP-exposed groups, throughout the 30-day DBP exposure period. This was expected since it has been established that spermatogenesis is particularly sensitive to several environmental toxicants in rats (Wong and Cheng 2011; Manfo et al. 2014). The quail testis has also been shown to be susceptible to the effects of some environmental toxicants (Aire 2005; Bello et al. 2014, 2019). Furthermore, our present findings corroborate similar observations in rodents, showing that most phthalates adversely affect testicular differentiation; as well as adult spermatogenesis in the adult rodent by provoking germ cell loss, testicular atrophy, and altered Leydig cell function (Ge et al. 2007; Spade et al. 2015). It should be noted that SEH was reduced regardless of the DBP doses, indicating a possible suppression of active spermatogenesis because it is known that most phthalates, including DBP, disrupt active spermatogenesis at several stages (Boekelheide 2005; Boekelheide et al. 2009). The thinning of the seminiferous epithelium and subsequent decrease in seminiferous tubular diameter was probably due to cell death (apoptosis) and the sloughing of germ cells (Alam et al. 2010a; Zhu et al. 2010). These studies corroborate the current findings that the sloughing of germ cells in the seminiferous epithelium resulted in reduced SEH, as morphometric alterations may have been a consequence of germ cell apoptosis (Ünal et al. 2013). In contrast, a recent in utero study showed that gestational exposure to high DBP dose altered seminiferous tubule morphometry and inhibited the proliferation of fetal testicular somatic cells, but did not affect apoptosis (Boekelheide et al. 2009). This observation may indicate that decreased proliferation, rather than increased apoptosis, is the underlying mechanism of altered fetal development of seminiferous tubules in DBP-exposed animals or birds. The present study has further demonstrated the potential of computer-assisted micro-stereological analysis in assessing the seminiferous tubular function that ordinarily is not possible to determine or quantify due to subtle morphological alterations that are not amenable to routine light microscopy. It has also been shown that the progressive morphometric alterations seen in the seminiferous epithelium, were, in fact, due to the direct toxic effects of DBP on testicular tubular histo-dynamics. These observed effects have been *dose-dependent* (as seen from the observations from cyto-architectural changes) and *parameter-specific, *resulting in the thinning of SEH, increase in diameter of tubular lumen, decreases in Leydig cell size.

Numerous in vivo mammalian studies have shown DBP to disrupt the androgen-regulated development of the male reproductive tract (Mylchreest and Foster 2000; Kavlock et al. 2002; Saillenfait et al. 2008; de Falco et al. 2015). Specifically, these results have indicated that phthalates may induce diverse adverse effects depending on the timing of exposure, the sex of the animal, and the critical developmental window. In certain phthalates such as DEHP, low doses administered at pre- and post-natal stage or to prepubertal and the adult rat was able to induce functional perturbations of Leydig, Sertoli, and germ cells (Akingbemi et al. 2007; Sharpe et al. 2003; Saillenfait et al. 2008; Alam et al. 2010a; Johnson et al. 2012; Walker et al. 2021). Likewise, higher doses of DBP (200, 400, and 600 mg/kg) exposure have been reported to induce testicular malformations in the male, resulting into seminiferous tubular necrosis and the absence of spermatogenesis in rats (Aly et al. 2016).

On the other hand, phthalates have been shown to interfere with the regulation of the hypothalamic-pituitary–gonadal (HPG) axis by alterations of gonadotropin-releasing hormone (GnRH), luteinizing hormone (LH), and follicle-stimulating hormone (FSH) (Jin and Yang 2014; Meltzer et al. 2015; Hlisníková et al. 2020), leading to downstream consequences on some steroidogenic enzymes and altered sex hormones. These findings suggest that DBP-induced changes are anti-androgenic, potentially suppressing Leydig cell steroidogenesis and active spermatogenesis. And when the present study is viewed in parallel with our previous report (Bello et al. 2014), showing DBP effects on enzyme genes that regulate Leydig cell steroidogenesis without changing cellular testosterone levels, the DBP effect on cyto-architectural ultrastructural alterations may compromise the structural integrity of the affected Leydig cells and thereby modifying the form (i.e., structural cell organelles) and ultimately the cell functions; and with attendant downstream reproductive consequences on the steroidogenic machinery (Alam and Kurohmaru 2021). For instance, the increased size and preponderance of electron-lucent lipid droplets or more precisely, “the cholesteryl esters” by Leydig cells implies that cholesterol utilization for androgen, such as testosterone biosynthesis was decreased. Thus, the absence of effects on serum testosterone levels, as reported previously by Bello et al. (2014), may have significantly been altered in the experimental animals due to one or both of two factors, namely, increased Leydig cell numbers arising from phthalate-induced proliferation (Akingbemi et al. 2001, 2004) or a decrease in testosterone degradation and excretion from the body (Eveillard et al. 2009).

In conclusion, the present findings have provided information on the deleterious effects of DBP in the testes of adult male Japanese quail exposed to DBP prepubertally, as well as validating techniques that are able to determine chemically-induced cellular alterations on Leydig cell steroidogenic function, from both micro-stereological and ultrastructural assessment perspectives.

## Data Availability

The data that support the findings of this study are available from the corresponding author (UMB), upon reasonable request.
